# Supplementary material for: Stepwise construction of dynamic microscale concentration gradients around hydrogel-encapsulated cells in a microfluidic perfusion culture device
Source: R Soc Open Sci. 2020 Jul 1;7(7):200027. doi: 10.1098/rsos.200027 (PMC7428233; doi:10.1098/rsos.200027)
Supplement: Electronic Supplementary Material [file rsos200027supp1.docx]

Electronic supplementary Material
Stepwise construction of dynamic microscale concentration gradients around hydrogel-encapsulated cells in a microfluidic perfusion culture device

Shinya Yamahira,^a^ Taku Satoh,^a^ Fumiki Yanagawa,^a^ Masato Tamura,^a^ Toshiyuki Takagi,^a^ Eri Nakatani,^a,b^ Yuta Kusama,^a,b^ Kimio Sumaru,^a^ Shinji Sugiura,^a^ and Toshiyuki Kanamori^a^

a. Biotechnology Research Institute for Drug Discovery, National Institute of Advanced Industrial Science and Technology (AIST), Tsukuba, Ibaraki, Japan.

b. Department of Bioengineering, Nagaoka University of Technology, Nagaoka, Niigata, Japan.

Fig. S1 An NMR spectrum of DBCO-PC-4armPEG.


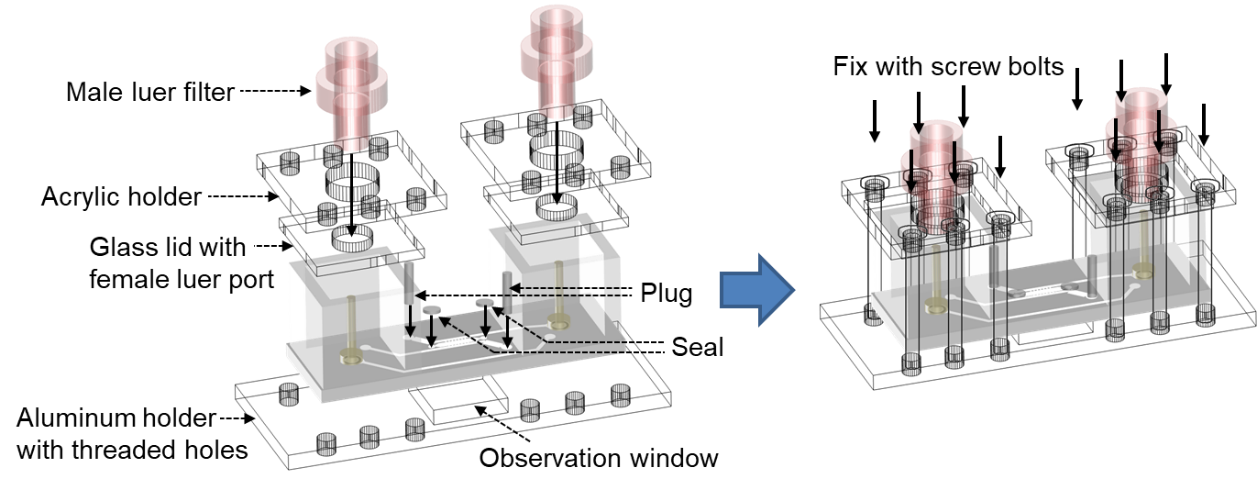


Fig. S2 Device assembling for perfusion culture.


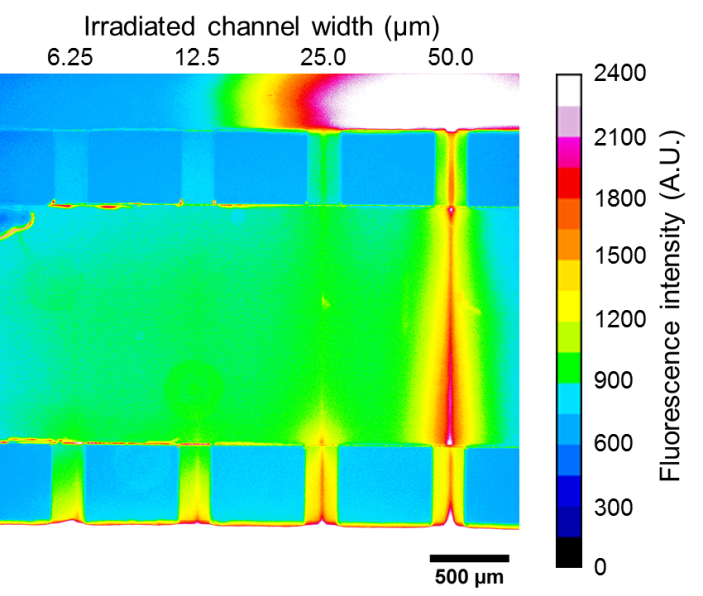


Fig. S3 Heat map of fluorescence intensity for the microchannels fabricated in the photodegradable hydrogel.


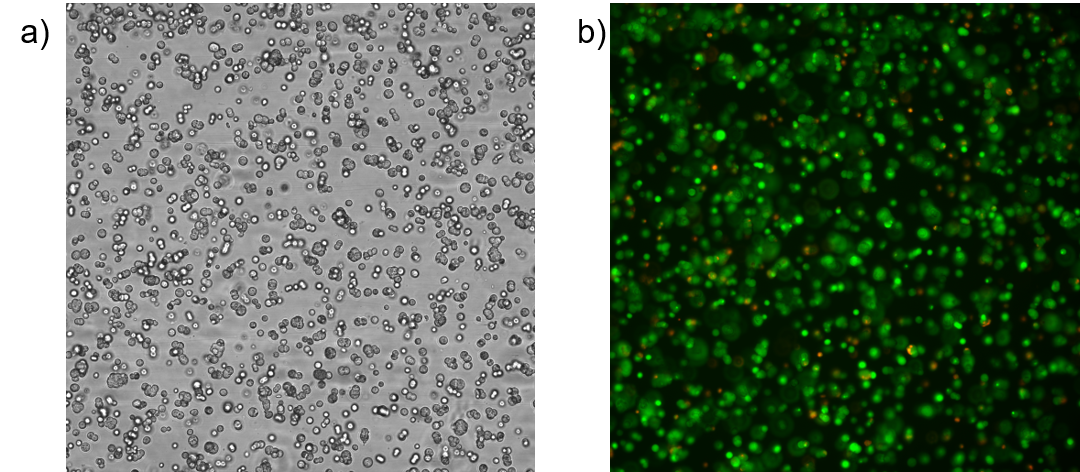


Fig. S4 Cell viability in the off-device hydrogel..


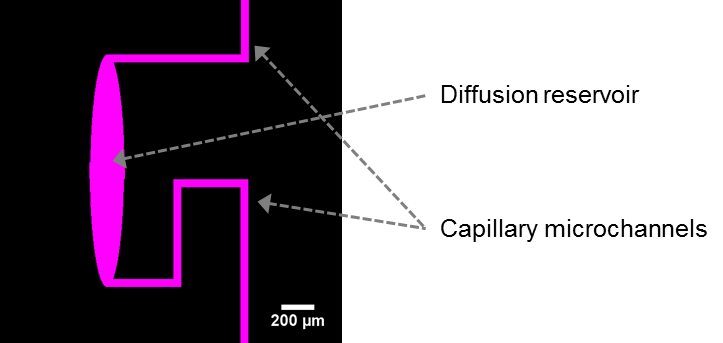


Fig. S5 Irradiated pattern for patterning of multi-molecular concentration gradients.


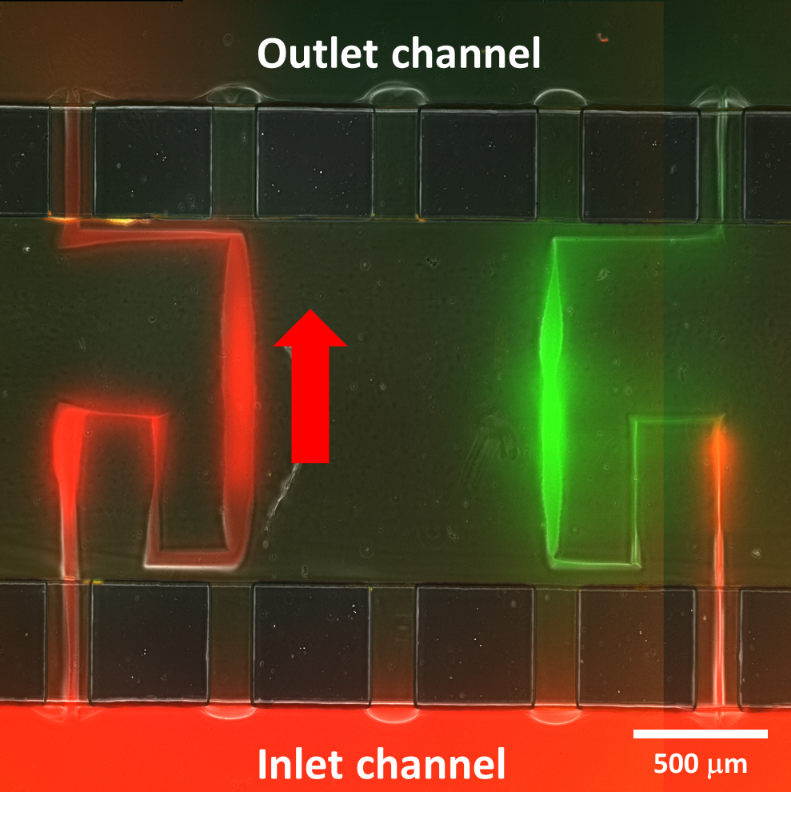


Fig. S6 After closing the first microchannel by expansion of the hydrogel by swelling, the Texas Red-dextran solution was introduced through the inlet channel. The solution flowed into only the second “diffusion reservoir”.


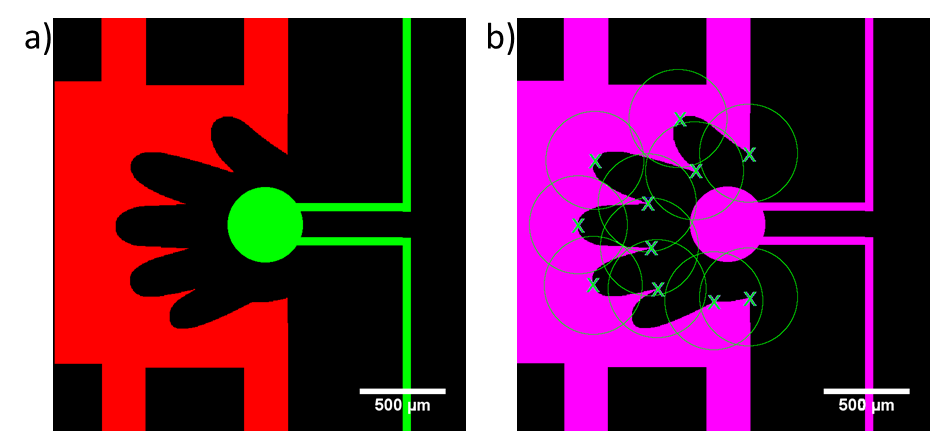


Fig. S7 Irradiated pattern for dynamic patterning of multi-molecular concentration gradients. a) Green and red patterns indicate irradiated patterns for the reservoir of FITC-Dextran solution and the flow path of Texas-Red dextran solution, respectively. b) X indicates the border points between the flow path and the gel with cells. Each green circle is centered on point X with a radius of 300 µm.
